# Neuropilins 1 and 2 mediate neointimal hyperplasia and re-endothelialization following arterial injury

**DOI:** 10.1093/cvr/cvv229

**Published:** 2015-09-26

**Authors:** Caroline Pellet-Many, Vedanta Mehta, Laura Fields, Marwa Mahmoud, Vanessa Lowe, Ian Evans, Jorge Ruivo, Ian Zachary

**Affiliations:** Division of Medicine, Centre for Cardiovascular Biology and Medicine, University College London, 5 University Street, London WC1E 6JF, UK

**Keywords:** Neuropilins, Smooth muscle cells, Endothelial cells, Neointimal hyperplasia, Re-endothelialization

## Abstract

**Aims:**

Neuropilins 1 and 2 (NRP1 and NRP2) play crucial roles in endothelial cell migration contributing to angiogenesis and vascular development. Both NRPs are also expressed by cultured vascular smooth muscle cells (VSMCs) and are implicated in VSMC migration stimulated by PDGF-BB, but it is unknown whether NRPs are relevant for VSMC function *in vivo*. We investigated the role of NRPs in the rat carotid balloon injury model, in which endothelial denudation and arterial stretch induce neointimal hyperplasia involving VSMC migration and proliferation.

**Methods and results:**

NRP1 and NRP2 mRNAs and proteins increased significantly following arterial injury, and immunofluorescent staining revealed neointimal NRP expression. Down-regulation of NRP1 and NRP2 using shRNA significantly reduced neointimal hyperplasia following injury. Furthermore, inhibition of NRP1 by adenovirally overexpressing a loss-of-function NRP1 mutant lacking the cytoplasmic domain (ΔC) reduced neointimal hyperplasia, whereas wild-type (WT) NRP1 had no effect. NRP-targeted shRNAs impaired, while overexpression of NRP1 WT and NRP1 ΔC enhanced, arterial re-endothelialization 14 days after injury. Knockdown of either NRP1 or NRP2 inhibited PDGF-BB-induced rat VSMC migration, whereas knockdown of NRP2, but not NRP1, reduced proliferation of cultured rat VSMC and neointimal VSMC *in vivo*. NRP knockdown also reduced the phosphorylation of PDGFα and PDGFβ receptors in rat VSMC, which mediate VSMC migration and proliferation.

**Conclusion:**

NRP1 and NRP2 play important roles in the regulation of neointimal hyperplasia *in vivo* by modulating VSMC migration (via NRP1 and NRP2) and proliferation (via NRP2), independently of the role of NRPs in re-endothelialization.

## 1. Introduction

Percutaneous transluminal coronary angioplasty (PTCA) continues to be used for coronary revascularization for emergency heart attack and for angina refractory to standard medical therapy, with ∼2 million procedures worldwide every year (www.ptca.org). The principal reason for failure of PTCA is restenosis, particularly in diabetics. A major underlying cause of restenosis, as well as of stenosis following coronary bypass and other vascular grafting, is the abnormal accumulation of neointimal vascular smooth muscle cells (VSMCs) resulting from media-to-intima migration and proliferation. These processes, together with deposition of extracellular matrix, lead to neointimal hyperplasia, causing luminal narrowing, thereby limiting the beneficial effects of intervention. The development of drug-eluting stents has considerably improved the outcome and is now the preferred method of revascularization, but restenosis remains an important cause for the failure of revascularization therapy.

Neuropilin 1 (NRP1) is a receptor for Class 3 semaphorins which regulate axonal guidance and neuronal patterning during embryogenesis,^1,2^ and for members of the VEGF family of angiogenic cytokines in endothelial cells, with an essential role in vascular development.^3,4^ NRPs are also increasingly recognized to be important mediators of other physiological and pathophysiological processes, including immunoregulation and tumourigenesis.^5^ NRP1 and the related molecule, NRP2, share a similar domain structure with a large multi-domain extracellular region essential for ligand binding, a single transmembrane domain and a small intracellular domain.^5^ NRP1 complexes with VEGF receptor 2 (VEGFR2) in the endothelium and mediates VEGF signalling important for directed cell migration, a function at least partly dependent on the NRP1 cytoplasmic domain.^6–9^ Furthermore, NRP1 is also able to mediate the cellular functions of non-canonical NRP1 ligands involving signalling and complexation with other growth factor receptors, including PDGF/PDGF receptors(R), fibroblast growth factor-2 (FGF-2),^10^ and transforming growth factor β (TGF-β) and its receptors.^11–16^ NRPs are highly expressed in VSMCs, mesenchymal stem cells (MSC), and hepatic stellate cells and mediate migration of these cells in response to PDGF by associating with PDGFRα and β, thereby regulating their phosphorylation state and downstream chemotactic signalling.^11,13,15,17^ It is so far unclear whether NRP1 plays a role in VSMC function *in vivo*. PDGF signalling is known to be a key regulator of VSMC migration following endovascular injury;^18^ PDGF-BB and its receptors, PDGFRα and PDGFRβ, are differentially up-regulated in a time-dependent manner after angioplasty.^19^ Other growth factors, such as FGF-2, are also strongly implicated in migration and proliferation of VSMC leading to neointima formation induced by endovascular injury.^20^ We examined whether NRPs play a role in neointimal remodelling induced by balloon angioplasty in the rat carotid artery, a well-characterized model of arterial remodelling following endothelial denudation. We found that endogenous NRP1 and NRP2 were up-regulated following arterial injury, and that disruption of NRP function either by overexpressing a NRP1 mutant lacking the cytoplasmic domain (NRP1 ΔC) or by targeted knockdown of NRP1 and NRP2, significantly reduced neointimal hyperplasia following arterial injury. Interestingly, NRP2 knockdown also selectively reduced neointimal VSMC proliferation and decreased the migration and proliferation of cultured VSMC, whereas NRP1 knockdown inhibited only VSMC migration. NRP1 and NRP2 inhibition also impaired re-endothelialization and reduced endothelial cell migration. These findings indicate novel roles for NRPs in mediating pathological VSMC accumulation, neointimal thickening, and endothelial regeneration in response to endovascular injury.

## 2. Materials and methods

An expanded Methods section is available in the Supplementary material online.

### 2.1 Rat carotid injury model, adenovirus treatments, and morphometry

All animal experiments were performed in compliance with the Animals (Scientific Procedures) Act (1986) and in accordance with Home Office and University College London guidelines. Animals were anaesthetized using a combination of Midazolam (625 μg/100 g) and Fentanyl (40 μg/100 g) supported by Halothan 0.5% at 2 L/min oxygen flow. Carotid injury in anaesthetized male Sprague-Dawley rats (Charles River UK) was performed using an embolectomy catheter as previously described (see Supplementary material online).^21^ Adenoviruses (Ad) were delivered to injured carotid arteries in pluronic^®^ gel F-127 (Sigma-Aldrich) containing 10^10^ adenoviral particles applied to the adventitial surface of the artery. Specimen retrieval was done following perfusion under terminal anaesthesia using pentobarbital via intraperitoneal injection. Two hundred millilitres of 0.9% saline solution was perfused at a rate of 20 mL/min via the abdominal aorta. The specimens were then carefully dissected through neck incision. Neointimal hyperplasia was assessed by determining the ratios of the intimal to medial areas (I/M) in haematoxylin- and eosin-stained paraffin-embedded sections using ImageJ.^22,23^ Re-endothelialization was assessed by immunohistochemical staining of endothelial nitric oxide synthase (eNOS)^24–26^ and also using Evan's blue staining of the whole artery.

### 2.2 Expression studies, cell culture, adenoviruses, and cell migration

Measurement of gene expression by absolute quantitative RT-PCR (qPCR) (see Supplementary material online, *Table S1* for primers), western blotting, immunofluorescent and immunohistochemical staining, generation of adenoviruses encoding NRP1 constructs and shRNAs targeted to NRP1 and NRP2, culture of rat aortic SMC (RAoSMC) and rat aortic endothelial cells (RAoEC), and assays of cell migration was performed as previously described^11,21–23,27–29^ (see expanded Methods section in the Supplementary material online).

### 2.3 Cell proliferation

Carotid artery neointimal proliferation was determined by bromodeoxyuridine (BrdU) labelling. BrdU solution (Sigma-Aldrich) was injected in rats intraperitoneally 24 h prior to tissue harvest. BrdU-labelled cells were detected by immunostaining paraffin-embedded arterial sections using anti-BrdU antibody (Dako, Glostrup, Germany). Proliferation of cultured RAoEC was determined in 96-well plates (seeding density of 7000 cells per well) by assessing cell confluence in living cells using an IncuCyte™ Zoom (Essen Bioscience) for up to 3 days.

### 2.4 Statistical analysis

Results are presented as means ± SEM. Statistical analysis was performed using GraphPad Prism Version 5 by using either one-way or two-way ANOVA with Bonferroni post tests, comparing a treatment with control conditions. Repeated-measures two-way ANOVA was made for cell proliferation assays and cell migration scratch assays performed using the IncuCyte™ Zoom (Essen Bioscience). The number of experiments or animals used in each study is specified for each figure. Statistical significance was validated at *P < 0.05.

## 3. Results

### 3.1 Neuropilins are up-regulated following angioplasty

**Figure 1:**
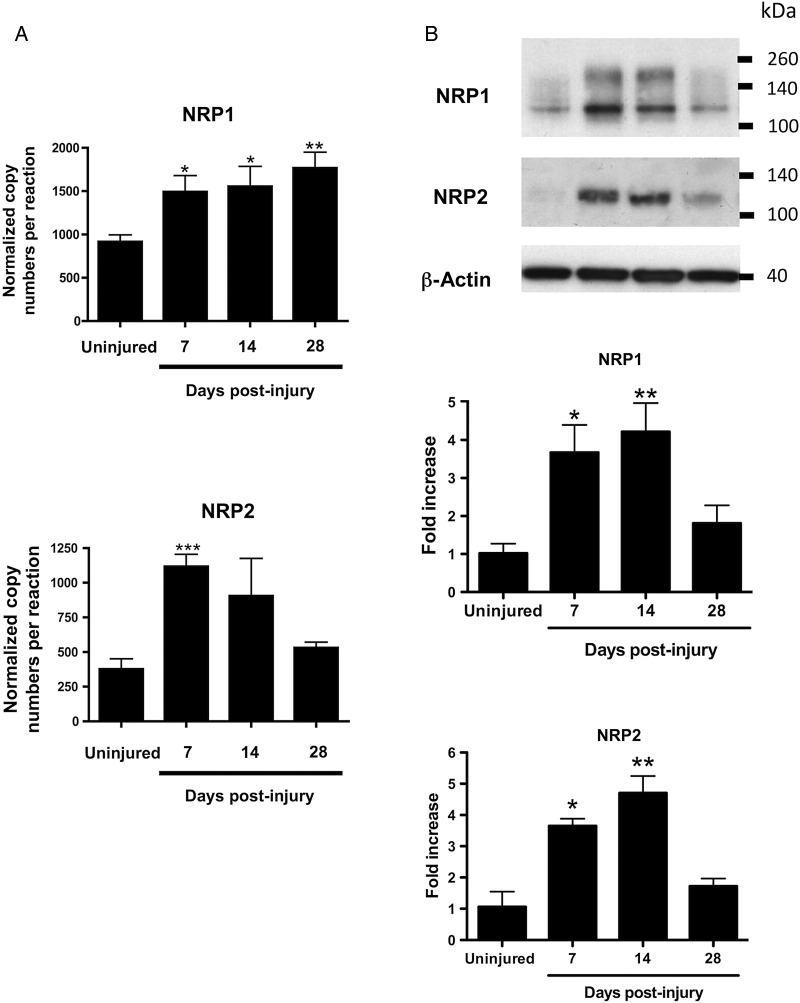
Neuropilins are up-regulated following carotid balloon angioplasty. Levels of NRP1 and NRP2 mRNA (*A*) and protein (*B*) expression were determined in uninjured rat carotid arteries and at different times after left carotid arterial balloon injury by absolute qPCR and western blot, respectively. Values for mRNA expression are means ± SEM; *n* = 6 arteries, *P < 0.05, **P < 0.01, ***P < 0.001. Protein expression was quantified by scanning densitometry of blots: means ± SEM; *n* = 4, *P < 0.05, **P < 0.01.

**Figure 2:**
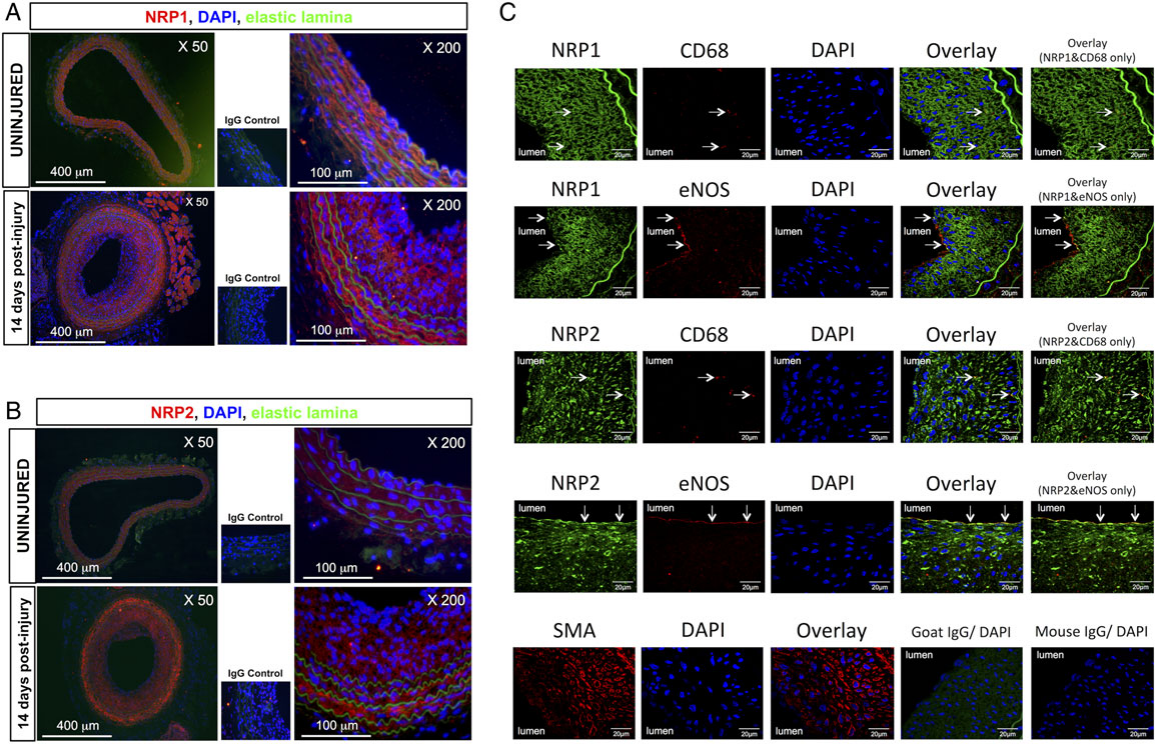
Neointimal expression of NRPs after balloon injury. (*A* and *B*) Representative IF images of carotid arteries showing endogenous expression of NRP1 (sc-7239, Santa Cruz) (*A*) and NRP2 (sc-13117, Santa Cruz) (*B*) following vascular injury in LCA (14 days after angioplasty) and in uninjured RCA (*n* = 3 for each treatment). Negative control staining with IgG and secondary antibody alone is shown in small photomicrographs at ×200 magnification. (*C*) LCA were harvested 28 days post-angioplasty and stained for either NRP1 (sc-7239, Santa Cruz), NRP2 (sc-54128, Santa Cruz), smooth muscle cell α actin (ab18460-1, Abcam^®^; SMA), CD68 (MCA341R, AbD Serotec^®^), or eNOS (610296, BD transduction laboratories™) as an endothelial cell marker. Co-staining of NRP1 and NRP2 with antibodies to eNOS and CD68 was performed on *n* = 6 arteries. White arrows indicate selected areas of colocalization of NRPs with eNOS and CD68. Negative control staining with appropriate IgG control antibodies with DAPI counterstaining is shown. The luminal side is placed as indicated on each panel, and the internal elastic lamina is visible by autofluorescence.

Assessment of NRP1 gene regulation during the development of neointimal hyperplasia by qPCR at 7, 14, and 28 days after injury of the left carotid artery (LCA) showed that NRP1 mRNA copy number was significantly increased at all time points in injured arteries compared with the uninjured right carotid arteries (RCA). NRP2 mRNA expression was also significantly elevated 7 days after angioplasty thereafter declining to control levels by 28 days after injury (*Figure 1A*). Assessment of VEGFR2 (Flk1) mRNA showed a nearly two-fold increase in the gene copy number 14 and 28 days after injury, but the mRNA copy number was very low relative to that of NRPs, and VEGFR2 up-regulation was not statistically significant (see Supplementary material online, *Figure S1*). VEGFR2 up-regulation probably reflects endothelial cell proliferation and repopulation of the lumen after injury. NRP1 and NRP2 protein expression increased significantly after injury with a maximum up-regulation at 7 and 14 days (*Figure 1B*). IF staining of arterial sections revealed expression of NRP1 and NRP2 after arterial injury in the medial and neointimal layers of the carotid 14 days after angioplasty (*Figure 2A* and *B*). Immunofluorescent staining of NRP1 and NRP2 showed extensive neointimal staining suggestive that NRP1 and NRP2 were at least partly localized to VSMC (*Figure 2C*). Immunostaining of VSMC with antibody to smooth muscle-specific α-actin (SMA) also showed extensive neointimal staining, though for technical reasons we were unable to quantify the extent of co-staining with NRPs. Some luminal cells were positive for NRP1 and NRP2 immunostaining, and these luminal cells showed co-staining for endothelial-specific eNOS (*Figure 2C*). It was further noted that sparse cells within the neointima were positive for both NRP1 and the monocyte/macrophage marker, CD68, as well as for NRP2 and CD68, indicating that some neointimal NRP positive cells were macrophages (*Figure 2C*). For NRP1, 5.9 and 2.7% immunofluorescent staining co-localized with eNOS and CD68, respectively, and corresponding values for NRP2 were 12.9 and 2.2%.

### 3.2 Neuropilins play a role in neointimal thickening induced by injury

**Figure 3:**
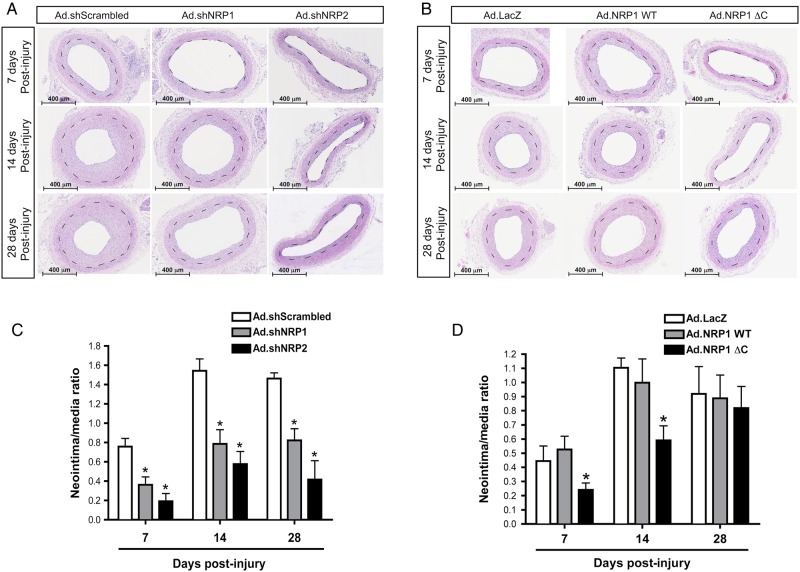
NRPs inhibition reduces neointima formation induced by balloon injury. Representative H&E pictures (*A* and *B*) and morphometric analysis (*C* and *D*) of left coronary arteries following arterial injury (7, 14, and 28 days post-injury) and delivery of adenoviruses encoding either shRNAs (*A* and *C*) or Ad.LacZ, Ad.NRP1 WT, and Ad.NRP1 ΔC (*B* and *D*). In *A* and *B*, dashed lines indicate the internal elastic lamina. In *C* and *D,* intima to media ratios were determined by morphometric analysis using Image J: means ± SEM; *n* = 6 animals per treatment at each time point, *P < 0.05.

The up-regulation of NRP1 and NRP2 in the carotid artery following injury suggested a possible role of NRPs in increased VSMC migration contributing to neointimal thickening and arterial remodelling. To address this possibility, we used two approaches: targeted knockdown of NRP expression using small hairpin (sh) RNAs specific for NRP1 and NRP2 and overexpression of adenoviral constructs encoding either a WT NRP1 or a mutant NRP1 lacking the cytoplasmic domain (NRP1 ΔC) previously shown to inhibit PDGF-BB-induced migration in human coronary artery VSMC and VEGF-induced endothelial cell migration (see Supplementary material online, *Figure S2*).^6,11,13^ Overexpression of Ad.shRNA constructs targeted to NRPs in carotid arteries significantly and selectively decreased NRP1 and NRP2 arterial mRNA and protein expression *in vivo* and protein expression in RAoSMC (see Supplementary material online, *Figure S3*). Morphometric analysis of the transduced vessels revealed that overexpression of either shNRP1 or shNRP2 significantly decreased neointimal hyperplasia, evaluated by determining I/M, compared with the shScrambled control at all time points (*Figure 3A* and *C* and see Supplementary material online, *Table S2*). NRP2 knockdown reduced neointima formation to a somewhat greater extent than NRP1 shRNA, though not significantly so (*P* = 0.1261). It was also examined whether combined knockdown of NRP1 and NRP2 could inhibit neointima formation more profoundly. The results of this experiment confirmed the inhibitory effects of targeted knockdown of either NRP alone, with NRP2 knockdown again exerting a marginally greater effect, but showed that the combined knockdown caused no greater inhibition than NRP2 shRNA alone (see Supplementary material online, *Figure S4*).

Delivery of Ad.NRP1 WT and Ad.NRP1 ΔC was performed in pluronic gel applied to the adventitial surface of carotid arteries at the time of injury as described previously.^30^ The efficiency of arterial gene transduction using this approach was demonstrated by examining expression of adenovirus-encoded Green Fluorescent Protein (Ad.GFP) and LacZ (Ad.LacZ) by PCR and immunostaining 7 days after injury and infection with vectors (see Supplementary material online, *Figure S5*). Adenovirally delivered GFP and LacZ were strongly expressed in injured rat carotid arteries, with substantial expression found in the media, adventitia, and neointima, as indicated by PCR (GFP only) (see Supplementary material online, *Figure S5A*) and immunohistochemical staining (see Supplementary material online, *Figure S5B*). At 7 and 14 days post-injury, protein expression of adenovirally delivered GFP or LacZ was detected, as indicated by western blot of whole carotid artery lysates, but expression decreased markedly at 28 days after injury (see Supplementary material online, *Figure S5C*).

Morphometric analysis of the vessels revealed that overexpression of the NRP1 ΔC mutant significantly decreased the formation of neointimal hyperplasia compared with the Ad.LacZ and Ad.NRP1 WT at 7 and 14 days post-injury (*Figure 3B* and *D*; see Supplementary material online, *Table S3*). At 28 days after injury, I/M ratios were modestly reduced compared with earlier times consistent with the observation that intimal hyperplasia reaches a peak after ∼2–3 weeks following injury and thereafter remains relatively constant.^31^ At this time point, there was no statistically significant difference between arteries transduced with LacZ, NRP1 WT, and NRP1 ΔC adenoviruses. The lack of a significant inhibitory effect of Ad.NRP1 ΔC at 28 days in part may reflect reduced adenoviral expression of this molecule at later times after delivery as indicated by LacZ and GFP expression (see Supplementary material online, *Figure S5C*).

### 3.3 Re-endothelialization and endothelial function

**Figure 4:**
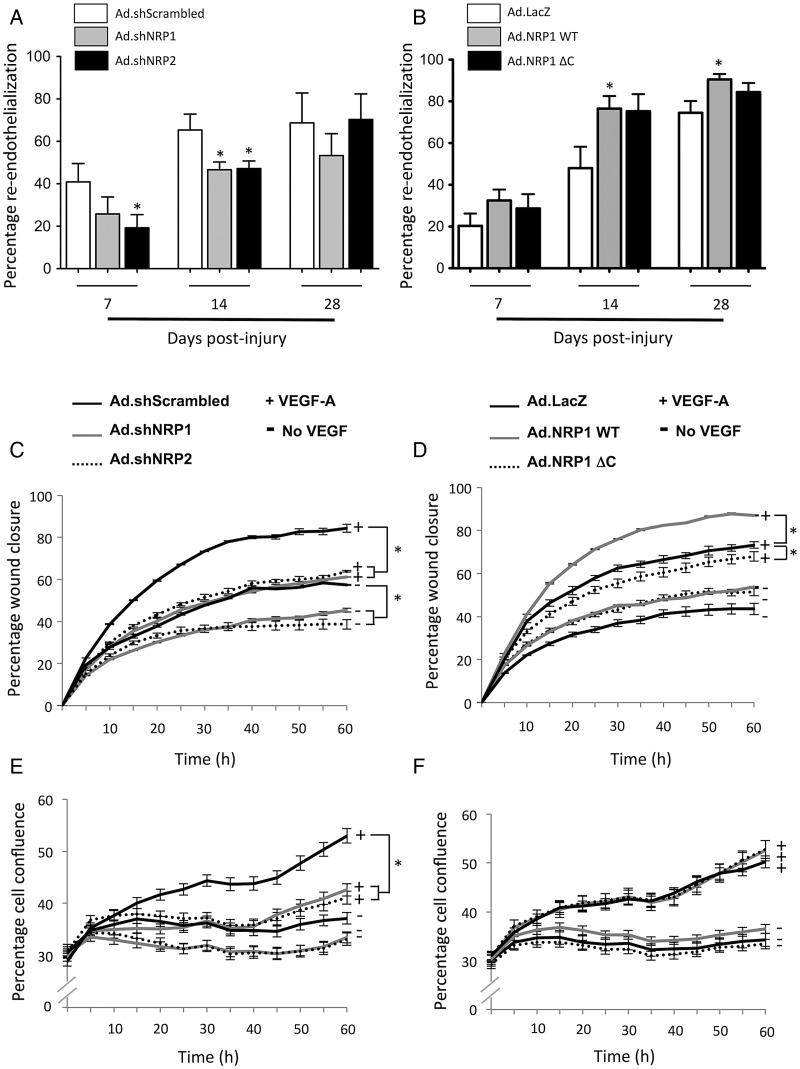
NRP inhibition impairs re-endothelialization following endothelial denudation. (*A* and *B*) Re-endothelialization was determined by quantification of eNOS immunostaining in balloon-injured carotid arteries 7, 14, and 28 days after injury and delivery of either (*A*), adenoviruses encoding shScrambled (open bars) or shRNAs targeted to NRP1 (grey bars) and NRP2 (black bars), or (*B*), Ad.LacZ (open bars), Ad.NRP1 WT (grey bars), and Ad.NRP1 ΔC (black bars), *n* = 6 animals per treatment at each time point, *P < 0.05. (*C–F*) Effects of adenoviruses encoding shScrambled (black line) or shRNAs targeted to NRP1 (grey line), NRP2 (dashed line) (*C* and *E*) or Ad.LacZ (black line), Ad.NRP1 WT (grey line), and Ad.NRP1 ΔC (black dashed line) (*D* and *F*) on VEGF-induced RAoEC migration (*C* and *D*) or RAoEC proliferation (*E* and *F*), each experiment counted 16 replicates and the data represent the average of three independent experiments, *P < 0.05.

Given that NRP1 has been shown to play an important role in endothelial cell migration, we investigated whether reduced neointimal hyperplasia consequent upon NRP knockdown or overexpression of NRP1 ΔC could be due to an effect on re-endothelialization. Endothelial luminal coverage was determined by immunostaining of eNOS at six different levels across the whole length of the injured artery. Continuous specific staining of the endothelium was observed in uninjured carotid arteries (see Supplementary material online, *Figure S6A*), whereas staining was almost completely absent immediately after injury (see Supplementary material online, *Figure S6C*) and progressively increased thereafter to ∼75% luminal coverage 28 days following injury (*Figure 4A* and *B*). Quantification of endothelial eNOS staining showed that endothelial coverage was significantly reduced by Ad.shRNA targeted to NRP1 at 14 days and by Ad.shRNA to NRP2 at 7 and 14 days, but it was not significantly affected by Ad.shRNA at 28 days (*Figure 4A*). In addition, *en face* staining of arteries using Evans blue dye at 14 days after injury also revealed decreased re-endothelialization of vessels following knockdown of NRP1 and NRP2, though this effect was significant only for Ad.shNRP1 vs*.* Ad.shScrambled (see Supplementary material online, *Figure S6G*). The reduced re-endothelialization values obtained from Evan's blue staining compared with immunohistochemical staining of eNOS may be due to leakage of dye in the mitotic-regenerating endothelium.^32^ Overexpression of Ad.NRP1 WT or Ad.NRP1 ΔC had no significant effect on re-endothelialization at 7 days, but at 14 days after injury, overexpression of both constructs enhanced re-endothelialization compared with control Ad.LacZ-transduced arteries (*Figure 4B*). Treatment with Ad.NRP1 WT also caused a significant enhancement in re-endothelialization at 28 days compared with Ad.LacZ (90 vs. 74%), while Ad.NRP1 ΔC had no significant effect at this time. Given these results, we evaluated effects of manipulating NRP expression on migration and proliferation in primary cultures of RAoEC in response to VEGF. Overexpression of Ad.NRP1 WT significantly enhanced VEGF-induced RAoEC migration (from 32 h onwards) and Ad.NRP1 ΔC significantly reduced RAoEC migration (from 48 h onwards), while overexpression of NRP1 WT and NRP1 ΔC did not alter RAoEC proliferation (*Figure 4D* and *F*). In contrast, Ad.shRNAs targeted to either NRP1 or NRP2 significantly inhibited RAoEC migration (from 28 and 32 h onwards for shNRP1 and shNRP2 vs. shScrambled, respectively) (*Figure 4C*) and proliferation (from 42 and 48 h onwards for shNRP1 and shNRP2 vs. shScrambled, respectively) (*Figure 4E*).

The effect of NRP knockdown on endothelial function of carotid arteries 14 days after angioplasty was assessed in organ bath studies of vascular reactivity. These experiments demonstrated a significantly reduced contractile response in the injured arteries compared with the uninjured arteries, but indicated no effect of Ad.shNRP1 or Ad.shNRP2, compared with Ad.shScrambled (see Supplementary material online, *Figure S7A*, *Table S4*). Relaxation of injured carotids was also significantly less than that of uninjured arteries. We observed a trend towards reduced relaxation in vessels treated with either Ad.shNRP1 or Ad.shNRP2 compared with Ad.shScrambled, but the differences were not statistically significant (see Supplementary material online, *Figure S7B*, *Table S5*).

### 3.4 Neointimal cell proliferation

Two major mechanisms could explain the inhibitory effects of NRP inhibition on neointima formation: inhibition of neointimal cell migration and/or reduced cell proliferation. Analysis of BrdU labelling in injured carotid arteries 7 days after angioplasty, a time when neointimal cell proliferation peaks in the rat carotid injury model,^31^ revealed abundant BrdU-labelled neointimal cells with sparser labelled cells present in the adventitia or media. Though this is likely to be due mainly to VSMC proliferation, we do not preclude that some BrdU labelling could arise from proliferation of inflammatory or endothelial cells. Although Ad.shRNAs targeting NRP1 and NRP2 both significantly reduced neointimal thickening (*Figure 3A*), Ad.shNRP1 had no significant effect on neointimal BrdU labelling, whereas Ad.shNRP2 caused a significant reduction in neointimal cell proliferation (see Supplementary material online, *Figure S8A*). Furthermore, neither Ad.NRP1 WT nor Ad.NRP1 ΔC had any significant effect on VSMC proliferation within neointimal lesions (see Supplementary material online, *Figure S8B*). *In vivo* proliferation was also assessed at 14 days post-injury, but very little proliferation was observed at this time (data not shown).

### 3.5 Role of neuropilins in migration and proliferation of RAoSMC

**Figure 5:**
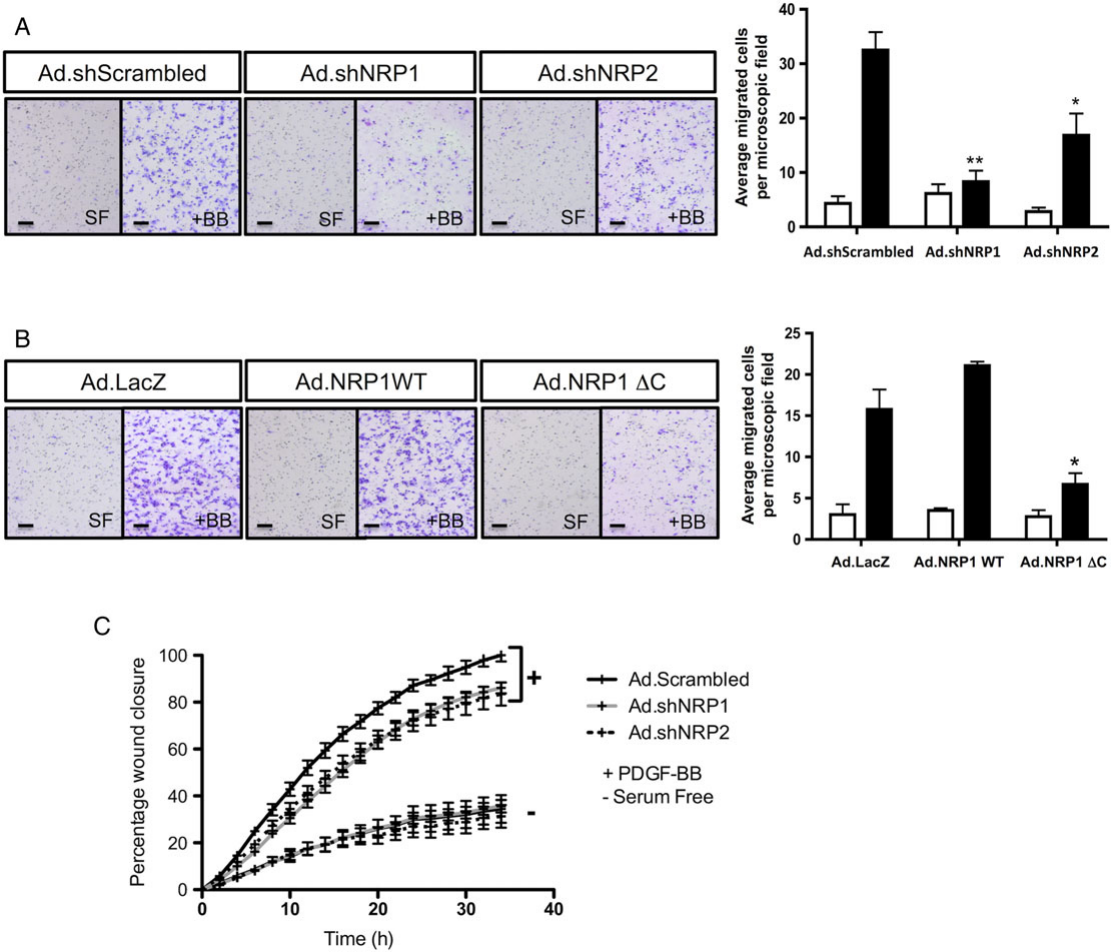
Inhibition of NRP1 and NRP2 reduces PDGF-BB-induced Rat VSMC chemotaxis. RAoSMC were infected either with NRP1, NRP2, and control Scrambled Ad.shRNAs (*A*) or with Adenoviruses encoding NRP1 WT, NRP1 ΔC, or LacZ (*B*), and after 24 h, infected cells were transferred to Transwell filters, and migration was determined in response to 30 ng/mL PDGF-BB (black bars), or no treatment (serum-free medium, open bars). The efficacy of NRP1 and NRP2 knockdown in RAoSMC was assessed by western blot (see Supplementary material online, *Figure S4C*). Representative Transwell filters are shown for each treatment (bar scale represents 100 μm). Quantification of migration is presented as means ± SEM from three independent experiments, and each treatment was performed in triplicate; *P < 0.05, ** *P* < 0.01. (*C*) RAoSMC in 96-well plates were infected either with NRP1, NRP2, and control Scrambled Ad.shRNAs for 48 h. Cells were then starved overnight before a precise scratch was generated using the WoundMaker™ (Essen BioScience). Migration was assessed in the presence or absence of 10 ng/mL PDGF-BB, using an IncuCyte ZOOM^®^ Live-Cell Imaging Platform. The graph represents three independent experiments: means ± SEM; *P < 0.05 (from 10 h onwards) for Ad.Scrambled vs*.* Ad.shNRP1. * *P* < 0.05 (from 14 h onwards) for Ad.Scrambled vs*.* Ad.shNRP2. Each treatment per experiment was performed in 16 replicates.

**Figure 6:**
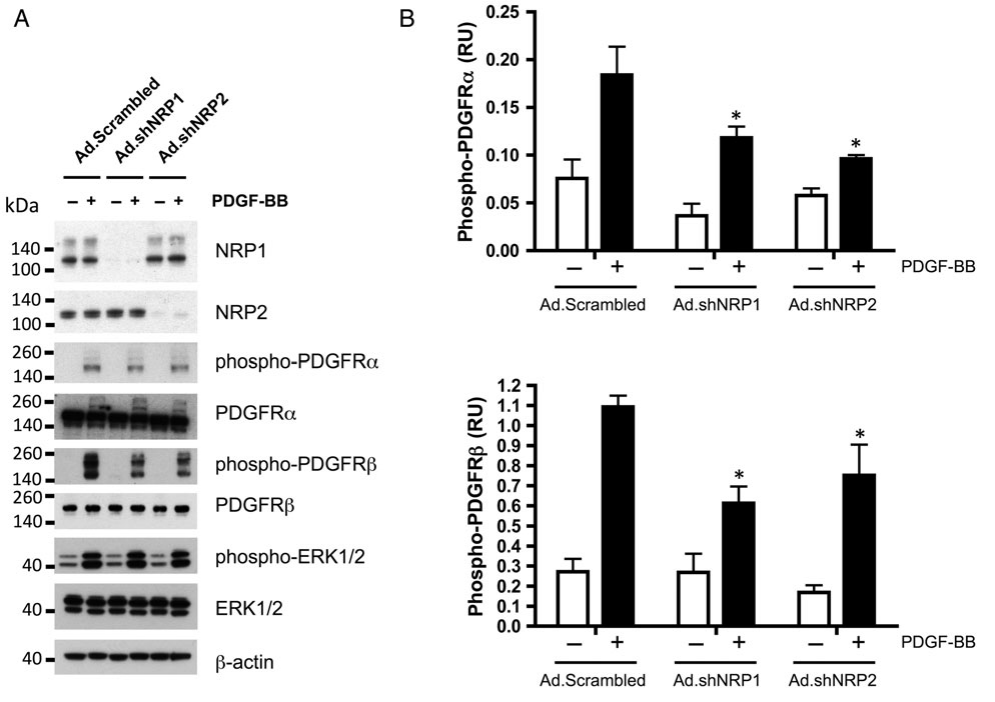
NRP1 and NRP2 knockdown reduces PDGF-BB-induced phosphorylation of PDGFRα and PDGFRβ in RAoSMC. RAoSMC were infected with NRP1, NRP2, or control Scrambled (Scr) Ad.shRNAs, and after 48 h, infected cells were starved in serum-free medium overnight before stimulation for 10 min with 30 ng/mL PDGF-BB (+, black bars), or with serum-free medium only (−, open bars). Lysates were immunoblotted as indicated (*A*), and data from three independent experiments were quantified (*B*, bars represent means ± SEM; *P < 0.05 vs. Ad.Scrambled + PDGF-BB).

Our results indicated that inhibition of NRP1 function in arterial VSMC by either targeted knockdown or overexpression of NRP1 ΔC had little effect on VSMC proliferation and, therefore, was likely to act primarily by inhibiting directed cell migration. This possibility was evaluated directly by examining the effect of shRNA-mediated knockdown of NRP1 and NRP2 on the migratory response of primary cultures of rat arterial SMC (RAoSMC) to PDGF-BB. Infection of RAoSMC with shRNAs targeted to rat NRP1 and NRP2 significantly reduced NRP protein expression in RAoSMC (*Figure 6*; see Supplementary material online, *Figure S3C*). NRP1 knockdown caused a significant decrease in cell migration in response to PDGF-BB, as determined in Transwell assays of chemotaxis and in scratch assays of wound closure (chemokinesis), while NRP2 shRNA also significantly inhibited the migratory response to PDGF-BB, although to a lesser extent (*Figure 5A* and *C*). We next examined the effects of adenoviral overexpression of NRP1 WT and the NRP1 ΔC mutant on PDGF-BB-induced RAoSMC migration (*Figure 5B*). Infection of RAoSMC with Ad.NRP1 WT did not significantly alter the migration of RAoSMC towards a gradient of PDGF-BB, compared with control cells infected with Ad.LacZ. In contrast, Ad.NRP1 ΔC infection caused a significant inhibition of cell migration induced by the PDGF-BB gradient (*Figure 5B*), indicating a role for the NRP1 cytoplasmic domain in downstream signalling mediating the chemotactic response of these cells, consistent with our previous findings in human VSMC.^11^ VSMC migration, as assessed by scratch assay, was not affected by the overexpression of the Ad.NRP1 WT or ΔC constructs (data not shown).

Analysis of neointimal BrdU labelling in rat carotid arteries indicated a role for NRP2 in VSMC proliferation. To test this directly, we compared the effects of NRP1 and NRP2 shRNAs on the proliferation of RAoSMC. Ad.shNRP1 had no effect on PDGF-BB-stimulated proliferation compared with Ad.shScrambled controls, whereas Ad.shNRP2 caused a small decrease in PDGF-BB-induced RAoSMC proliferation (data not shown).

### 3.6 Regulation of PDGF signalling in rat VSMC by neuropilins

The mechanisms mediating the role of NRPs in VSMC migration and proliferation were addressed by investigating the effects of NRP knockdown and adenoviral overexpression on PDGF receptor activation in RAoSMC. As shown in *Figure 6*, PDGF treatment of RAoSMC induced a marked increase in tyrosine phosphorylation of PDGFRβ and PDGFRα. NRP1- and NRP2-targeted knockdown using shRNA infection in these cells significantly decreased PDGFRα and PDGFRβ tyrosine phosphorylation without affecting total PDGFR levels (*Figure 6*). Adenoviral overexpression of NRP1 WT or NRP1 ΔC had no significant effect on phosphorylation of either PDGFRβ or PDGFRα in RAoSMC (results not shown).

## 4. Discussion

NRPs are expressed in arterial VSMC and have been shown to mediate PDGF signalling and migration in VSMC. NRP1 is also reported to be most strongly expressed in late mouse embryos in the VSMC of large vessels^33^ and in VSMC as well as endothelial cells in human coronary arteries and aortae used for coronary artery bypass grafting.^34^ However, hitherto, the role of NRPs in VSMC functions *in vivo* has not been investigated. A major conclusion of the present study is that NRP1 and NRP2 are both expressed in the VSMC of the carotid artery wall and are significantly up-regulated at mRNA and protein levels in the carotid artery after endothelial balloon injury. Both NRPs were most strongly expressed at 7 and 14 days post-injury, coincident with the most active phase of neointimal VSMC accumulation and with the maximum extent of neointimal thickening in this model. Further consistent with the notion that NRPs are involved in neointimal VSMC accumulation, immunofluorescent staining revealed strong neointimal NRP1 and NRP2 expression in the neointima further suggestive of expression in neointimal VSMC. It was noted that whereas total arterial NRP1 and NRP2 protein expression declined at 28 days, NRP1 mRNA expression remained elevated at this time, perhaps reflecting a later phase of NRP1 expression in the endothelial repopulation of injured arteries.

The second important conclusion of this paper is that NRP1 and NRP2 both contribute to neointimal VSMC hyperplasia and neointima formation in the injured rat carotid injury model. This conclusion is based on two major lines of evidence: (i) targeted shRNAs to either NRP1 or NRP2, effective in reducing NRP expression in cultured rat VSMC and in the carotid artery, significantly inhibited neointimal thickening at 7, 14, and 28 days after endothelial balloon injury; (ii) adenoviral overexpression of an NRP1 ΔC mutant lacking the cytoplasmic domain, previously shown to inhibit VEGF- and PDGF-BB-induced migration of, respectively, endothelial cells and VSMC, also strongly inhibited neointima formation at 7 and 14 days after injury. Given that NRP inhibition using siRNA- or shRNA-mediated NRP1 and NRP2 knockdown, or by overexpression of NRP1 ΔC, inhibits PDGF-BB-induced migration of cultured primary human^11^ and rat arterial VSMC, we propose that the inhibitory effect of interfering with NRP function on neointima formation results primarily from inhibition of VSMC migration, probably due to inhibition of PDGF-BB signalling, though whether PDGF signalling is the only or major target for the action of NRP1 inhibition is not clear. For example, NRP1 also regulates signalling by TGF-β and its receptors, which are important mediators of VSMC proliferation.^15,35,36^ The inhibitory effect of Ad.NRP1 ΔC on neointimal hyperplasia is most likely due to a dominant negative effect of this construct on NRP1 functions in VSMC dependent on its cytoplasmic domain, as we previously demonstrated in human VSMC.^11^ A role for the NRP1 cytoplasmic domain in arteriogenesis has been demonstrated in mice with a global knock-in of NRP1 lacking its cytoplasmic region. These mice exhibit normal developmental angiogenesis, but impaired neonatal arteriogenesis in the heart, kidney, and hindlimb.^37^ The NRP1 cytoplasmic domain is thought to function at least in part through interaction of its C-terminal PDZ domain binding motif to the PDZ domain protein, synectin, and this and other possible protein interactions with the NRP1 cytoplasmic domain could play a scaffolding role in cell migration.^5,37^ The role of the NRP2 cytoplasmic domain is less clear and warrants further investigation. A key role for NRP1 specifically in neointimal VSMC migration is also inferred from the finding that neither NRP1 shRNA nor NRP1 ΔC caused inhibition of neointimal cell proliferation measured by *in vivo* BrdU labelling. NRP1 knockdown also had no effect on RAoSMC proliferation. Furthermore, previously published work indicates an important role for NRP1 in directed cell migration induced by chemotactic cues such as PDGF-BB and VEGF, but that NRP1 is unimportant for cell proliferation.^4–9,11,38^ While we cannot preclude NRP1 cytoplasmic domain playing a role in other cellular processes contributing to neointimal hyperplasia, our present findings strongly indicate that inhibition of NRP1 function in the rat carotid artery selectively interferes with VSMC migration underlying neointimal VSMC accumulation.

In contrast to NRP1, the cellular functions of NRP2 are less well understood. Our finding that shRNA-mediated NRP2 knockdown inhibited neointima formation indicates a novel role for NRP2 in pathological vascular remodelling. Since NRP2 knockdown also inhibited PDGF-BB-induced chemotaxis of human^11^ and rat VSMC, our results are consistent with NRP2 playing a role in directed VSMC migration contributing to neointimal expansion. This may be mediated either independently of NRP1 by, for example, a direct interaction between NRP2 and PDGFRα or β, and/or could arise from an interaction between NRP1 and NRP2. Our previous findings indicate that NRP1 and NRP2 readily associate with each other, consistent with the conclusion that these molecules heterodimerize or form mixed oligomers.^9^ The finding that the combination of shRNAs to NRP1 and NRP2 had no additive inhibitory effect on neointima formation suggests that the two NRPs lie on the same pathway. Since Ad.shNRP2 also significantly reduced *in vivo* neointimal BrdU labelling and caused a small decrease in RAoSMC proliferation, NRP2 may also contribute to the proliferation as well as migration of VSMC, though further work is required to support this conclusion.

The mechanism through which NRPs regulate PDGF-induced VSMC chemotaxis has not been fully defined yet, but previous findings show that NRP1 knockdown inhibits PDGF-induced PDGFRα activation.^11,13,15^ Herein we demonstrate that NRP1 and NRP2 knockdown significantly reduced PDGFRα and PDGFRβ tyrosine phosphorylation in rat VSMC, consistent with our previous findings that NRP1 knockdown inhibited PDGFR activation in human coronary artery VSMC.^11^ These results suggest that impaired PDGFR activation consequent upon NRP knockdown contributes to the inhibitory effects of NRP knockdown on VSMC migration *in vivo* after carotid injury and in cultured VSMC. In contrast to the effects of NRP1 knockdown, overexpression of NRP1 ΔC had no significant effect on PDGFR activity, consonant with our previous finding that this mutant had no effect on activation of PDGFR or VEGFR in U87 glioma cells,^6^ and indicating that the NRP1 cytosolic domain is not required for PDGFR activity in rat VSMC. The differential roles of NRP1 and NRP2 in VSMC proliferation, and the underlying mechanisms involved, warrant further study.

In the rat balloon injury model, endothelial denudation triggers vascular remodelling, resulting in rapid neointima formation due to the proliferation and migration of VSMC, which is followed by a slower regeneration of the endothelium. In this latter process, based on genetic and biochemical evidence pointing to a key role of endothelial NRP1 in angiogenesis and directed endothelial cell migration,^4–9^ NRP1 is hypothesized to be essential for endothelial cell migration from contiguous uninjured endothelium. This study is the first to examine the role of NRPs in endothelial regeneration of a large artery and demonstrates that targeted depletion of NRP1 or NRP2 using Ad.shRNAs markedly reduced re-endothelialization at 7 and 14 days after balloon injury. The retardation of re-endothelialization caused by NRP1 and NRP2 knockdowns is likely mediated by inhibition of endothelial cell migration and proliferation, a conclusion supported by the finding that shRNAs targeted to NRP1 and NRP2 also reduced RAoEC migration and proliferation. Conversely, overexpression of NRP1 WT significantly enhanced endothelial re-coverage 14 and 28 days after endothelial denudation and also augmented RAoEC migration. These data are consistent with a large body of evidence supporting a role for NRPs in mediating endothelial cell migration *in vivo* and in cells, and support the conclusion that NRP1 and NRP2 play a significant role in re-endothelialization.

Reduced re-endothelialization is predicted to enhance neointima formation, whereas knockdown of NRPs strikingly decreased the I/M ratio. This finding strongly argues that the inhibitory effect of NRP1 and NRP2 depletion on neointima formation is independent of the role of NRPs in re-endothelialization and is instead mediated via a direct inhibitory effect on VSMC migration. Furthermore, the inhibitory effects of Ad.NRP1 ΔC on neointimal hyperplasia at 7 days after injury are unlikely to be due to its effects on re-endothelialization, since it had no significant effect on re-endothelialization at this time.

In contrast, increased re-endothelialization is expected to reduce neointimal hyperplasia, but Ad.NRP1 WT did not have such an effect. We propose that this is because the enhancement of re-endothelialization caused by Ad.NRP1 WT may not be sufficient to significantly impact upon neointimal thickening. The trend towards enhancement of re-endothelialization by Ad.NRP1 ΔC was unexpected, since previous studies showed that this construct inhibited migration of HUVECs.^6,11^ However, though Ad.NRP1 ΔC significantly reduced RAoEC migration, it had no significant effect on RAoEC proliferation. These data suggest that endothelial cell proliferation and enhancement of re-endothelialization by overexpression of NRP1 are largely dependent on the NRP1 extracellular domain and do not require the intracellular domain. In addition, re-endothelialization *in vivo* relies more on endothelial proliferation, and consequently, inhibition of RAoEC migration by Ad.NRP1 ΔC may not impact so significantly upon re-endothelialization. The significant reduction in neointimal thickening caused by Ad.NRP1 ΔC is likely due to its direct inhibitory effects on VSMC migration. Studies of endothelial contractility in injured carotid arteries showed reduced responses following injury consistent with previous findings,^39^ but showed no effect of NRP1 or NRP2 knockdown on the contractile response mediated via α_1_-adrenegric receptor stimulation. We observed a tendency towards attenuated endothelium-dependent relaxation in the carotid arteries treated with Ad.shNRP1/2 compared with Ad.shScrambled, which, though not significant, is consistent with our finding of reduced re-endothelialization in response to NRP knockdowns.

These findings argue that a major role of NRP1 and NRP2 in early arterial remodelling following endothelial damage is as a mediator of VSMC migration contributing to neointimal expansion. NRPs are required for optimum endothelial regeneration, as indicated by the inhibitory effects of NRP knockdown on re-endothelialization, but this effect does not appear to be strong enough to counteract the inhibitory effect of targeted shRNAs on neointimal growth. This novel role for NRP1 and NRP2 in mediating pathological arterial neointimal hyperplasia in the rat injured carotid artery model suggests that these receptors may be suitable targets for novel therapies designed to prevent or reduce excessive VSMC accumulation in vascular proliferative disorders. In particular, the ability of NRP1 ΔC to both reduce neointimal hyperplasia, without impairing and even modestly enhancing re-endothelialization, suggests that selective targeting of the NRP1 C-terminal domain could reduce neointimal hyperplasia while sparing endothelial function.

## Supplementary material

Supplementary material is available at *Cardiovascular Research* online.

## Funding

This work was supported by British Heart Foundation (BHF) programme grant RG/06/003 (I.E. and M.M.) and BHF Project grant PG/12/65/29840 (to I.Z. and C.P-M.), a BHF studentship (to V.L.), and by funding from the European Union's Seventh Program for research, technological development and demonstration under grant agreement no. 278313, ‘BIOMAGSCAR’ (V.M. and L.F.).

## References

[1] He Z, Tessier-Lavigne M Neuropilin is a receptor for the axonal chemorepellent Semaphorin III. *Cell* 1997;90:739–751. 9288753 10.1016/s0092-8674(00)80534-6

[2] Kitsukawa T, Shimizu M, Sanbo M, Hirata T, Taniguchi M, Bekku Y, Yagi T, Fujisawa H Neuropilin-semaphorin III/D-mediated chemorepulsive signals play a crucial role in peripheral nerve projection in mice. *Neuron* 1997;19:995–1005. 9390514 10.1016/s0896-6273(00)80392-x

[3] Soker S, Takashima S, Miao HQ, Neufeld G, Klagsbrun M Neuropilin-1 is expressed by endothelial and tumor cells as an isoform-specific receptor for vascular endothelial growth factor. *Cell* 1998;92:735–745. 9529250 10.1016/s0092-8674(00)81402-6

[4] Gu C, Rodriguez ER, Reimert DV, Shu T, Fritzsch B, Richards LJ, Kolodkin AL, Ginty DD Neuropilin-1 conveys semaphorin and VEGF signaling during neural and cardiovascular development. *Dev Cell* 2003;5:45–57. 12852851 10.1016/s1534-5807(03)00169-2 PMC3918747

[5] Pellet-Many C, Frankel P, Jia H, Zachary I Neuropilins: structure, function and role in disease. *Biochem J* 2008;411:211–226. 18363553 10.1042/BJ20071639

[6] Evans IM, Yamaji M, Britton G, Pellet-Many C, Lockie C, Zachary IC, Frankel P Neuropilin-1 signaling through p130Cas tyrosine phosphorylation is essential for growth factor-dependent migration of glioma and endothelial cells. *Mol Cell Biol* 2011;31:1174–1185. 21245381 10.1128/MCB.00903-10 PMC3067908

[7] Wang L, Zeng H, Wang P, Soker S, Mukhopadhyay D Neuropilin-1-mediated vascular permeability factor/vascular endothelial growth factor-dependent endothelial cell migration. *J Biol Chem* 2003;278:48848–48860. 14514674 10.1074/jbc.M310047200

[8] Kawamura H, Li X, Goishi K, van Meeteren LA, Jakobsson L, Cebe-Suarez S, Shimizu A, Edholm D, Ballmer-Hofer K, Kjellen L, Klagsbrun M, Claesson-Welsh L Neuropilin-1 in regulation of VEGF-induced activation of p38MAPK and endothelial cell organization. *Blood* 2008;112:3638–3649. 18664627 10.1182/blood-2007-12-125856 PMC2572791

[9] Herzog B, Pellet-Many C, Britton G, Hartzoulakis B, Zachary IC VEGF binding to NRP1 is essential for VEGF stimulation of endothelial cell migration, complex formation between NRP1 and VEGFR2, and signaling via FAK Tyr407 phosphorylation. *Mol Biol Cell* 2011;22:2766–2776. 21653826 10.1091/mbc.E09-12-1061 PMC3145551

[10] West DC, Rees CG, Duchesne L, Patey SJ, Terry CJ, Turnbull JE, Delehedde M, Heegaard CW, Allain F, Vanpouille C, Ron D, Fernig DG Interactions of multiple heparin binding growth factors with neuropilin-1 and potentiation of the activity of fibroblast growth factor-2. *J Biol Chem* 2005;280:13457–13464. 15695515 10.1074/jbc.M410924200

[11] Pellet-Many C, Frankel P, Evans IM, Herzog B, Junemann-Ramirez M, Zachary IC Neuropilin-1 mediates PDGF stimulation of vascular smooth muscle cell migration and signalling via p130Cas. *Biochem J* 2011;435:609–618. 21306301 10.1042/BJ20100580 PMC3086270

[12] Banerjee S, Sengupta K, Dhar K, Mehta S, D'Amore PA, Dhar G, Banerjee SK Breast cancer cells secreted platelet-derived growth factor-induced motility of vascular smooth muscle cells is mediated through neuropilin-1. *Mol Carcinog* 2006;45:871–880. 16847823 10.1002/mc.20248

[13] Ball SG, Bayley C, Shuttleworth CA, Kielty CM Neuropilin-1 regulates platelet-derived growth factor receptor signalling in mesenchymal stem cells. *Biochem J* 2010;427:29–40. 20102335 10.1042/BJ20091512 PMC3441150

[14] Glinka Y, Prud'homme GJ Neuropilin-1 is a receptor for transforming growth factor beta-1, activates its latent form, and promotes regulatory T cell activity. *J Leukoc Biol* 2008;84:302–310. 18436584 10.1189/jlb.0208090 PMC2504713

[15] Cao S, Yaqoob U, Das A, Shergill U, Jagavelu K, Huebert RC, Routray C, Abdelmoneim S, Vasdev M, Leof E, Charlton M, Watts RJ, Mukhopadhyay D, Shah VH Neuropilin-1 promotes cirrhosis of the rodent and human liver by enhancing PDGF/TGF-beta signaling in hepatic stellate cells. *J Clin Invest* 2010;120:2379–2394. 20577048 10.1172/JCI41203 PMC2898590

[16] Glinka Y, Stoilova S, Mohammed N, Prud'homme GJ Neuropilin-1 exerts co-receptor function for TGF-beta-1 on the membrane of cancer cells and enhances responses to both latent and active TGF-beta. *Carcinogenesis* 2011;32:613–621. 21186301 10.1093/carcin/bgq281

[17] Liu W, Parikh AA, Stoeltzing O, Fan F, McCarty MF, Wey J, Hicklin DJ, Ellis LM Upregulation of neuropilin-1 by basic fibroblast growth factor enhances vascular smooth muscle cell migration in response to VEGF. *Cytokine* 2005;32:206–212. 16289960 10.1016/j.cyto.2005.09.009

[18] Ferns GA, Raines EW, Sprugel KH, Motani AS, Reidy MA, Ross R Inhibition of neointimal smooth muscle accumulation after angioplasty by an antibody to PDGF. *Science* 1991;253:1129–1132. 1653454 10.1126/science.1653454

[19] Majesky MW, Reidy MA, Bowen-Pope DF, Hart CE, Wilcox JN, Schwartz SM PDGF ligand and receptor gene expression during repair of arterial injury. *J Cell Biol* 1990;111:2149–2158. 2172262 10.1083/jcb.111.5.2149 PMC2116329

[20] Francis DJ, Parish CR, McGarry M, Santiago FS, Lowe HC, Brown KJ, Bingley JA, Hayward IP, Cowden WB, Campbell JH, Campbell GR, Chesterman CN, Khachigian LM Blockade of vascular smooth muscle cell proliferation and intimal thickening after balloon injury by the sulfated oligosaccharide PI-88: phosphomannopentaose sulfate directly binds FGF-2, blocks cellular signaling, and inhibits proliferation. *Circ Res* 2003;92:e70–e77. 12690039 10.1161/01.RES.0000071345.76095.07

[21] Santiago FS, Ishii H, Shafi S, Khurana R, Kanellakis P, Bhindi R, Ramirez MJ, Bobik A, Martin JF, Chesterman CN, Zachary IC, Khachigian LM Yin Yang-1 inhibits vascular smooth muscle cell growth and intimal thickening by repressing p21WAF1/Cip1 transcription and p21WAF1/Cip1-Cdk4-cyclin D1 assembly. *Circ Res* 2007;101:146–155. 17556661 10.1161/CIRCRESAHA.106.145235

[22] Khurana R, Moons L, Shafi S, Luttun A, Collen D, Martin JF, Carmeliet P, Zachary IC Placental growth factor promotes atherosclerotic intimal thickening and macrophage accumulation. *Circulation* 2005;111:2828–2836. 15911697 10.1161/CIRCULATIONAHA.104.495887

[23] Khurana R, Zhuang Z, Bhardwaj S, Murakami M, De ME, Yla-Herttuala S, Ferrara N, Martin JF, Zachary I, Simons M Angiogenesis-dependent and independent phases of intimal hyperplasia. *Circulation* 2004;110:2436–2443. 15477408 10.1161/01.CIR.0000145138.25577.F1

[24] Noiseux N, Boucher CH, Cartier R, Sirois MG Bolus endovascular PDGFR-beta antisense treatment suppressed intimal hyperplasia in a rat carotid injury model. *Circulation* 2000;102:1330–1336. 10982551 10.1161/01.cir.102.11.1330

[25] Indolfi C, Torella D, Coppola C, Curcio A, Rodriguez F, Bilancio A, Leccia A, Arcucci O, Falco M, Leosco D, Chiariello M Physical training increases eNOS vascular expression and activity and reduces restenosis after balloon angioplasty or arterial stenting in rats. *Circ Res* 2002;91:1190–1197. 12480821 10.1161/01.res.0000046233.94299.d6

[26] Torella D, Leosco D, Indolfi C, Curcio A, Coppola C, Ellison GM, Russo VG, Torella M, Li Volti G, Rengo F, Chiariello M Aging exacerbates negative remodeling and impairs endothelial regeneration after balloon injury. *Am J Physiol Heart Circ Physiol* 2004;287:H2850–H2860. 15231505 10.1152/ajpheart.01119.2003

[27] Fantin A, Herzog B, Mahmoud M, Yamaji M, Plein A, Denti L, Ruhrberg C, Zachary I Neuropilin 1 (NRP1) hypomorphism combined with defective VEGF-A binding reveals novel roles for NRP1 in developmental and pathological angiogenesis. *Development* 2014;141:556–562. 24401374 10.1242/dev.103028 PMC3899814

[28] Vandesompele J, De Preter K, Pattyn F, Poppe B, Van Roy N, De Paepe A, Speleman F Accurate normalization of real-time quantitative RT-PCR data by geometric averaging of multiple internal control genes. *Genome Biol* 2002;3:RESEARCH0034. 12184808 10.1186/gb-2002-3-7-research0034 PMC126239

[29] Bustin SA, Benes V, Garson JA, Hellemans J, Huggett J, Kubista M, Mueller R, Nolan T, Pfaffl MW, Shipley GL, Vandesompele J, Wittwer CT The MIQE guidelines: minimum information for publication of quantitative real-time PCR experiments. *Clin Chem* 2009;55:611–622. 19246619 10.1373/clinchem.2008.112797

[30] Siow RC, Mallawaarachchi CM, Weissberg PL Migration of adventitial myofibroblasts following vascular balloon injury: insights from in vivo gene transfer to rat carotid arteries. *Cardiovasc Res* 2003;59:212–221. 12829192 10.1016/s0008-6363(03)00292-x

[31] Clowes AW, Clowes MM Kinetics of cellular proliferation after arterial injury. II. Inhibition of smooth muscle growth by heparin. *Lab Invest* 1985;52:611–616. 4040189

[32] Chien S, Lin SJ, Weinbaum S, Lee MM, Jan KM The role of arterial endothelial cell mitosis in macromolecular permeability. *Adv Exp Med Biol* 1988;242:59–73. 3245515 10.1007/978-1-4684-8935-4_8

[33] Jubb AM, Strickland LA, Liu SD, Mak J, Schmidt M, Koeppen H Neuropilin-1 expression in cancer and development. *J Pathol* 2012;226:50–60. 22025255 10.1002/path.2989

[34] Alattar M, Jiang C, Luan Z, Pan T, Liu L, Li J Neuropilin 1 expression in human aortas, coronaries and the main bypass grafts. *Eur J Cardiothorac Surg* 2014;46:967–973. 24722942 10.1093/ejcts/ezu118

[35] Khan R, Agrotis A, Bobik A Understanding the role of transforming growth factor-beta1 in intimal thickening after vascular injury. *Cardiovasc Res* 2007;74:223–234. 17349984 10.1016/j.cardiores.2007.02.012

[36] Majesky MW, Lindner V, Twardzik DR, Schwartz SM, Reidy MA Production of transforming growth factor beta 1 during repair of arterial injury. *J Clin Invest* 1991;88:904–910. 1832175 10.1172/JCI115393 PMC295478

[37] Lanahan A, Zhang X, Fantin A, Zhuang Z, Rivera-Molina F, Speichinger K, Prahst C, Zhang J, Wang Y, Davis G, Toomre D, Ruhrberg C, Simons M The neuropilin 1 cytoplasmic domain is required for VEGF-A-dependent arteriogenesis. *Dev Cell* 2013;25:156–168. 23639442 10.1016/j.devcel.2013.03.019 PMC3774154

[38] Pan Q, Chanthery Y, Liang WC, Stawicki S, Mak J, Rathore N, Tong RK, Kowalski J, Yee SF, Pacheco G, Ross S, Cheng Z, Le CJ, Plowman G, Peale F, Koch AW, Wu Y, Bagri A, Tessier-Lavigne M, Watts RJ Blocking neuropilin-1 function has an additive effect with anti-VEGF to inhibit tumor growth. *Cancer Cell* 2007;11:53–67. 17222790 10.1016/j.ccr.2006.10.018

[39] Antonaccio MJ, Normandin D, Ferrer P Reduced contractile function after balloon denudation of rat carotid arteries. *Eur J Pharmacol* 1994;256:17–21. 8026560 10.1016/0014-2999(94)90610-6

